# Deep learning for small and big data in psychiatry

**DOI:** 10.1038/s41386-020-0767-z

**Published:** 2020-07-15

**Authors:** Georgia Koppe, Andreas Meyer-Lindenberg, Daniel Durstewitz

**Affiliations:** 1 Department of Theoretical Neuroscience, Central Institute of Mental Health, Medical Faculty Mannheim, Heidelberg University, Square J5, 68159 Mannheim, Germany; 2 Department of Psychiatry and Psychotherapy, Central Institute of Mental Health, Medical Faculty Mannheim, Heidelberg University, Square J5, 68159 Mannheim, Germany

**Keywords:** Diagnostic markers, Predictive markers, Translational research, Psychiatric disorders, Diseases of the nervous system

## Abstract

Psychiatry today must gain a better understanding of the common and distinct pathophysiological mechanisms underlying psychiatric disorders in order to deliver more effective, person-tailored treatments. To this end, it appears that the analysis of ‘small’ experimental samples using conventional statistical approaches has largely failed to capture the heterogeneity underlying psychiatric phenotypes. Modern algorithms and approaches from machine learning, particularly deep learning, provide new hope to address these issues given their outstanding prediction performance in other disciplines. The strength of deep learning algorithms is that they can implement very complicated, and in principle arbitrary predictor-response mappings efficiently. This power comes at a cost, the need for large training (and test) samples to infer the (sometimes over millions of) model parameters. This appears to be at odds with the as yet rather ‘small’ samples available in psychiatric human research to date (*n* < 10,000), and the ambition of predicting treatment at the single subject level (*n* = 1). Here, we aim at giving a comprehensive overview on how we can yet use such models for prediction in psychiatry. We review how machine learning approaches compare to more traditional statistical hypothesis-driven approaches, how their complexity relates to the need of large sample sizes, and what we can do to optimally use these powerful techniques in psychiatric neuroscience.

## Introduction

Current diagnostic and prognostic schemes in psychiatry need improvement. While current diagnostic methods are optimized for reliability, the underlying neurobiology is complex and variable [1, 2], both because the etiology of psychiatric disorders is highly diverse [3], and because the brain and behavior are per se highly complex systems involving multiple levels of temporal and spatial granularity and millions of nonlinear feedback loops. It has been argued that a diverse array of biophysical and biochemical factors may give rise to similar functionality at the level of the neuronal dynamics underlying behavior and, vice versa, that the same changes in neural dynamics may produce different behavioral outputs depending on the context [4]. These observations may partly explain why only a subgroup of patients respond to drug or psychotherapeutic treatment approved for any given disorder [5–7]. Personalized forms of therapy therefore require a different characterization that supplements categorical conventional diagnoses. This approach could be based on the analysis of large patient cohorts that include sufficient heterogeneity covering a wider range of personal disease histories, and on trans-diagnostic and multi-level approaches to the identification of pathological mechanisms underlying mental illness [8], that is, on the integration of many different data modalities [9–11], from genetic and molecular information (‘omics’) to brain and behavioral data. On the other hand, individual subject-level information needs to be integrated with such an account when designing personalized therapies.

There have been high hopes recently that artificial intelligence (AI) algorithms, in particular from the field of deep learning (DL), can meet these challenges. DL algorithms excel in processing highly complex data within which data features may interact at multiple levels and in highly nonlinear ways. In consequence, when combined with large amounts of data, they may have an enormous potential for healthcare services (see [9] for review). For instance, deep neural networks (DNNs) are remarkably successful at tasks requiring object or scene recognition [12–14] and natural language processing [15, 16]. DNNs have shown human to super-human performance in challenging board games by inferring rules mostly from ‘own experience’, playing the game against themselves, rather than from expert knowledge [17]. This ability to automatically learn relevant higher-level representations from raw data, also referred to as automatic feature extraction, is one central aspect which makes the application of DNNs in biomedical areas attractive [9, 18, 19]. DNNs have for instance already been successfully adopted to automatize skin and breast cancer detection [20, 21]. In the sector of mental health, recent studies have begun to harness the potential of DNNs and ‘big data’, especially in domains which are particularly data-rich, such as online social media platforms or smartphone and mobile sensor based data (e.g., [22–26]). Attempts to collect big data in other data domains, including for instance data on brain structure and function, genetics, or behavior on cognitive tasks, have been actively pushed by different consortia and funders (e.g., ENIGMA, ABIDE, ADNI, ADHD-200, OASIS, ABCD).

Building big multi-modal data bases is certainly an important step in identifying coherent patient subgroups in an unsupervised manner, gaining a better mechanistic understanding by acknowledging interactions and connections between different levels of analysis, and for personalizing treatments. But how much data do we need, when is ‘big’ big enough? Scientific data sets are often relatively small, carefully harvested in thoughtfully designed experiments, even when many of them are combined into common data bases. Even methods that generate a high volume of data, such as transcriptomics or neuroimaging, are often applied to a limited number of human subjects. Can DNNs efficiently be used on such comparatively small data bases? And how is the apparent conflict resolved between the need to process huge data sets on the one hand side, yet to construct subject-level models taking very individual information into account on the other hand? The present article attempts to address some of these questions, from a statistical and machine learning (ML) perspective, and discusses some of the factors that play a role both on the data side, as well as on the side of the models used for analysis.

## Models in statistics and ML

As discussed in the previous section, psychiatric research needs to address a variety of related challenges, including the identification of biomarkers for robust diagnostics, the identification of subgroups with shared disease characteristics (biological and psychological features) and common therapeutic response profiles, and personalization of treatments through subject-level predictions of potential outcomes and disease trajectories. These efforts are underpinned by the search for a deeper understanding of the neurobiological mechanisms underlying aberrant cognitive and emotional function across disorders, and the design of effective medication and intervention strategies based upon these insights (e.g., [10, 27]). From a statistical point of view, the former set of challenges may be formulated in terms of regression or classification problems, or in terms of unsupervised detection of structure (clustering) [28], while the latter, deeper scientific, questions may be supported by combining statistical and ML techniques with computational modeling [29, 30].

For a classification problem, for instance, we may want to predict treatment responses or symptom severity from brain activation during a cognitive task, or to distinguish between individuals diagnosed with different psychiatric disorders based on structural features of the brain (e.g., [31]). In both of these cases we can express our problem in terms of a relationship between an output variable $y$ (e.g., clinical diagnosis), or a set of output variables $y$ (e.g., severity of different symptoms), and a set of input variables or features ***x*** (e.g., functional activation in different brain areas). In principle, these input variables or features may come from different modalities (e.g., measures of structural connectivity vs. functional activation vs. polygenetic risk scores). If the outputs $y$ are categorical class labels, e.g., clinical diagnoses, we call this a classification problem, while a regression problem refers to the situation where outcomes $y$ are continuously (real-) valued, or at least bear ordinal relationships (like the natural numbers) as, e.g., ratings on scales of symptom severity. Both regression and classification problems are examples of supervised settings, for which not just the input data ***x*** but also the outputs ***y*** are known for a so-called training set.

In contrast, if, for instance, we question current diagnostic schemes and would like to identify novel types of clinically relevant groupings in feature space ***x*** unbiased by current nosological knowledge, we call this an unsupervised setting, a domain of ML and computational modeling.

In both types of settings we often formulate the problem in terms of a mathematical model of the data, either in terms of a functional relationship *f*_*θ*_ between ***x*** and ***y*** in the supervised case, where *θ* denotes parameters of this function (e.g., regression weights), or just in terms of the data itself in the unsupervised case. An (point) estimate of this function (denoted $\hat f$), or more specifically of its parameters (denoted $\hat \theta$), is obtained by changing these parameters such that some form of loss function is minimized (or some optimality criterion is maximized), a process called model training or, in statistical terms, model estimation or inference (in Bayesian inference, we would seek to determine the full [posterior] distribution across parameters *θ*, not just a point estimate). Such a loss function could be, for instance, the mean-squared-error (MSE) in the Gaussian case, i.e., the average sum of squared deviations between true and predicted outputs, or the negative log-likelihood of the data, which quantifies how likely it is to observe the current data given some estimate of the parameters. In the unsupervised case, a loss function may, for instance, be some type of measure that formalizes an idea of structure in the data, e.g., specifying the between-group vs. within-group distances for any possible assignment of data points to groups.

### Hypothesis testing vs. prediction

**Fig. 1:**
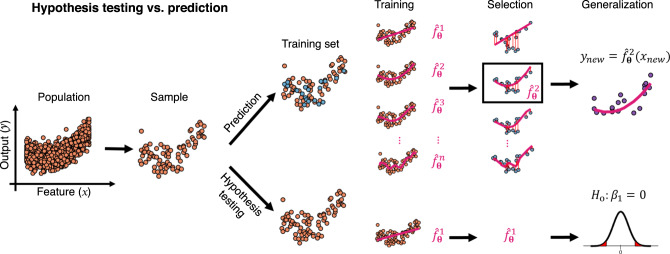
Statistical hypothesis testing vs. prediction in machine learning. The philosophy in classical statistical hypothesis testing (bottom path) is to draw a random sample from a population and estimate parameters of a model, which is assumed to describe the population sufficiently well (hence does not need to be selected among a larger class of models). Hypotheses about the population are then tested in terms of the model parameters. For instance, one may test whether there is a linear relationship between a feature and an output by formulating a null hypothesis on the slope parameter *β*_1_. In prediction, in contrast, which is what most machine learning methods aim for, we should look for the model which is the best in predicting outcomes in new samples (purple dots). Hence, rather than settling on one model a priori which is believed to describe the statistical properties of the true population, multiple models are trained in order to select the one which minimizes the loss on an independent validation set (blue dots).

There is no principal difference between models in statistics and ML, and both can be used, in principle, for either hypothesis testing or prediction (see Fig. 1). This distinction between hypothesis testing and prediction, however, is indeed important: while traditionally statistics has been more concerned with hypothesis testing, machine learners have been more interested in prediction [32]. In classical statistical hypothesis testing, we evaluate a probabilistic statement about the data, often formulated in terms of parameters of the model (e.g., that certain regression coefficients are equal to zero), and aim to obtain a probability for how likely a certain state of affairs (related to the null hypothesis) holds in the whole population of potential observations (which may be finite or infinite) given the model assumptions. This probabilistic inference is based on the observed training data alone. In prediction, in contrast, we aim to forecast future previously unobserved outcomes, e.g., the likely output $y$^(*new*)^ given a new observation ***x***^(*new*)^ (also called test data if used to formally evaluate the prediction error (PE), see Section ‘Model complexity, sample size, and generalization’).

Another, related issue here is whether the stated models are probabilistic or deterministic: For hypothesis testing at some level there are always random variables and probability distributions involved, such that relationships between input and output variables, for instance, are formulated in terms of moments of probability distributions, e.g., $\mu _y := E\left[ {y{\mathrm{|}}{\boldsymbol{x}}} \right] = f_\theta ({\boldsymbol{x}})$, where *f*_*θ*_ is the function that maps variables ***x*** onto the conditional mean (expectation value) $\mu_{y}$ of the distribution of $y$, and *θ* are its parameters (e.g., regression coefficients). For prediction, the function *f*_*θ*_ does not necessarily have to express a probabilistic relationship, i.e., we may just have $y$ = *f*_*θ*_(***x***), expressing outcomes $y$ directly as some (deterministic) function of the features ***x***. However, in modern ML probabilistic models are getting more and more popular (sometimes termed statistical ML), as they also provide a sense of the uncertainty associated with predictions. Although this enables formal hypothesis testing in principle as well, these models and their associated probability distributions are often tedious and difficult to handle.

Finally, while in statistics the functions or models *f*_*θ*_ are usually quite simple and/or allow for precise and unique analytical solutions, meaning that we can obtain an exact and unique solution to the optimization problem through ‘paper & pencil’ derivations, in ML the functional relationship *f*_*θ*_ may be quite complex, like a DNN. While the latter are potentially much more powerful in detecting and utilizing complex, higher-order nonlinear feature combinations for prediction, they are unfortunately also often much less interpretable than if *f*_*θ*_ were a simple linear function.

### DNNs and the universal function approximation theorem (UAT)

**Fig. 2:**
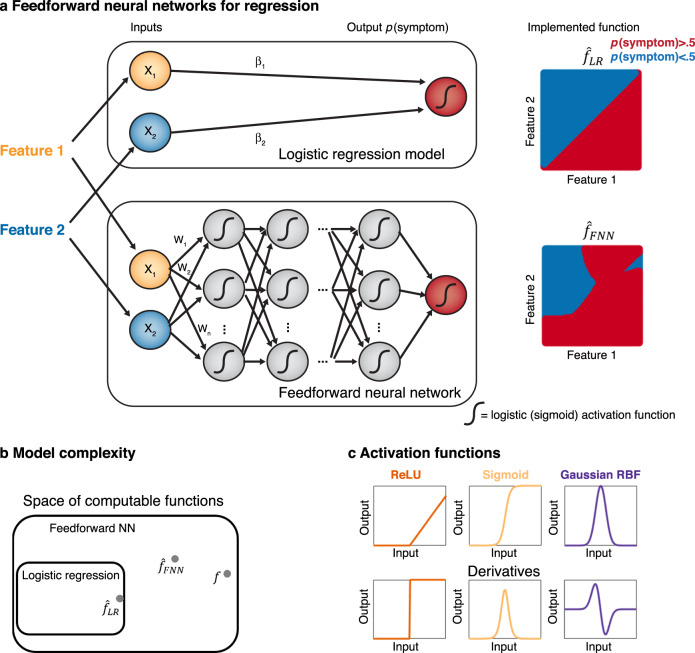
Feed-forward neural networks (FNNs) and function approximation. **a** Schematic of a logistic regression model (top) and FNN model (bottom) to predict an output (here symptom probability) from two types of features (e.g., brain function and structure). While the logistic regression model directly maps the weighted inputs through a logistic (sigmoid) type function, the FNN first filters the weighted inputs in successive stages by propagating them through multiple layers of units, with a nonlinear, e.g., sigmoid, activation function. While the logistic regression model can only separate two features linearly, the recombination of inputs across multiple stages allows the FNN to implement quite complex (in fact, arbitrarily complex) input output mappings (right panel). **b** In other words, the FNN has a much larger space of functions it can implement and thus a higher model complexity, including logistic regression functions as special cases, and may therefore be able to infer a function (denoted by $\hat f_{\rm{FNN}}$) closer to the true function *f*. **c** FNNs can be constructed with different types of activation functions such as ReLUs, sigmoids, or radial basis functions (RBFs; top panel). A strength of ReLUs is that their derivative is piecewise constant, whereas sigmoids and RBFs may have strongly varying gradients and saturate at the extremes (bottom panel).

DNNs likely constitute the most powerful class of ML models, at least from a mathematical-computational perspective, and in their most basic and most commonly employed form are deterministic. In visual terms, they can be understood as networks of artificial neurons, units, or nodes arranged in a feed-forward chain of layers, termed a feed-forward neural network (FNN; Fig. 2a), with each node computing some nonlinear function *f* (the so-called activation function) on the weighted sum of its inputs. Symbolically, this corresponds to a function *f*_*θ*_ that may be written as a deep nesting of multiple nonlinear functions $y = f(f\left( {f\left( {f \ldots f\left( {\boldsymbol{x}} \right) \ldots } \right)} \right))$ arranged in a chain.

In a first stage, an input layer receives the information about the inputs or predictors ***x*** (e.g., regional gray volume, or connectivity between areas [33–35]), which is then propagated forward via ‘synaptic’ connections with specific connection weights through one or multiple hidden layers, up to an output layer which represents the prediction $\hat y$ of the true (but unknown) outcome $y$. A neural network (NN) is often called ‘shallow’ if it contains just one or two hidden layers, and ‘deep’ if there are more of them. A DNN is trained by adjusting all its connection weights (the model’s parameters *θ*) such that the error between predicted ($\hat y$) and true ($y$) outcomes is minimized across a training set for which the true $y$ are known (for reviews see [12, 15, 36]), a process in which successive hidden layers of the network tend to learn more and more abstract representations of the data (e.g., edges and corners on early layers for visual images and fully segmented object representations on deeper layers, cf. [13, 37, 38]), much like the ventral visual processing stream of the human brain [39]. There are several different types of DNN architectures and models, such as multi-layer perceptrons, convolutional neural networks (CNN), or deep-belief networks, to some of which we will return below (see [36]).

Often in neuroscience and psychiatry we deal with sequential or time series data, where either sequences of inputs and outputs may have to be mapped onto each other (as in language), or some informative characteristics of the temporal structure are to be extracted. Not only do measurements in psychiatric and neuroscientific research often come as time series, e.g., in the form of functional magnetic resonance imaging (fMRI), electroencephalography (EEG), or mobile sampling data, or as sequential behavioral responses across trials of an experiment, but mental illness is a temporally dynamic and evolving phenomenon per se [4, 40], with quite heterogeneous temporal trajectories across individuals [3]. Just like classical statistics extends the class of regression models to the time series domain by also regressing values ***y*** onto their own past, as in auto-regressive moving-average (ARMA) models or ARMA models with exogenous inputs (ARMAX), NNs can be extended to the time series domain by incorporating previous function outputs, ${\boldsymbol{y}}_t = f_\theta ({\boldsymbol{y}}_{t - 1},{\boldsymbol{x}}_t)$. These devices are called recurrent neural networks (RNN), since they do not only include feed forward but also recurrent connections between units, i.e., activity may propagate back and forward among units. This means activity can reverberate in RNN just as in the real brain, and they can produce sequences of outputs completely on their own, like giving answers to questions as in common virtual assistants. Mathematically, RNN constitute discrete-time dynamical systems and they come with a whole set of novel properties that pure feed-forward NNs lack (see, e.g., [4]). Some researchers refer with ‘depth’ in RNN more to their temporal depth (in contrast to the ‘spatial depth’, i.e., the number of layers), by which one means the temporal lags or the time scales across which dependencies among observations and temporal structure can be detected by the system [41]. Some in this sense, deep RNN architectures have been purpose designed to bridge long temporal delays, such as long short-term memory (LSTM) [15] or gated recurrent unit (GRU)-based networks [42].

NNs with just one nonlinear hidden layer have a surprising mathematical property that all simpler statistical models, like the classes of general or generalized linear models, lack: In principle they can represent or approximate arbitrarily closely any continuously valued function $y$ = *f*(***x***) between predictors and outcomes, according to the much celebrated universal approximation theorem (UAT) due to Cybenko [43], Hornik et al. [44], and Funahashi [45] (and similar theorems exist for non-continuous mappings, like binary outcomes; [46]). That is, whatever the true underlying functional relation $y$ = *f*(***x***) in the real data is, a NN with just one hidden layer would be able to represent it (see Fig. 2a)! This of course raises the question why including more than one hidden layer, as in DNNs, is a sensible thing to do. It turns out that shallow NNs with just one layer and deep NNs fundamentally differ in how the number of units required to approximate a given function grows with the required accuracy of the approximation: While under some conditions the number of units required to achieve a given level of accuracy may only grow algebraically in the number of layers, exponentially more units may be required within a given layer to achieve that same level [47–50]. Besides these computational reasons, it has been observed that DNNs are capable of representation learning or automatic feature extraction, i.e., can construct the most useful representations of data themselves directly from the raw data across successive layers. For instance, when trained on facial images, a DNN will learn to represent simple features such as edges and nodes in early layers, then eyes and noses in later layers, and finally entire faces [37]. Without prior knowledge the model identifies noses and eyes as predictive features of faces.

We conclude by pointing out that similar theorems as for function representation in feed-forward NNs exist for RNN as well: RNN can approximate, in principle, arbitrarily closely any dynamical system that may have generated the true time series observed [45, 51, 52], and could represent any Turing machine [53]. While these theorems ascertain that in principle any feed-forward or time-dependent (dynamical) function could be implemented in terms of NNs with as little as one hidden layer, they make no statements about the difficulties involved in finding that implementation, or how much data are required to achieve an approximation of satisfying accuracy. Increasing the number of units or layers, or more generally the complexity of the function *f*_*θ*_, will enable to approximate more complicated functions to the degree of accuracy desired, but it will generally also increase the sample size needed for model estimation or training. This is because model complexity and sample size are intimately related, as we will discuss next.

## Model complexity, sample size, and generalization

**Fig. 3:**
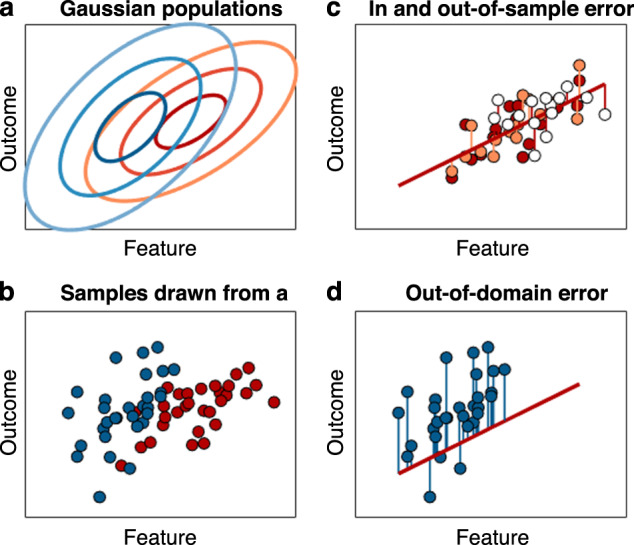
Different types of prediction errors. **a** Contours of two Gaussian distributions associated with two fictional populations (red and blue) showing a probabilistic relationship between a feature and an outcome (e.g., brain volume reduction and age). The ellipses mark points of equal probability density at standard deviations *σ* = 1, 2, 3, indicating the spread of the Gaussians. The red population shows slightly less spread (potentially related to stricter inclusion criteria or differences in the measurement device used for this population). **b** Two random samples of *n* = 30 points drawn from both distributions (indicated by corresponding colors). **c** 50% of the red sample (depicted in B) is used to fit a linear model (thick skewed red line). The remaining 50% of sample points (the test set), here displayed as white circles, are used to evaluate the *out-of-sample* error (red vertical lines). Another sample of outcomes is drawn at the exact same feature values used for training (orange circles) and used to evaluate the *in-sample* prediction error (orange vertical lines). **d** The model (red line, same as in **c**) is now employed to predict the outcome for the blue (more broad) sample (potentially collected at a different site). The blue vertical lines mark the *out-of-domain* prediction error. This error appears to be larger than both the other errors (**c**) and indicates a systematic underestimation of the outcome.

In healthcare, when we try to determine a diagnosis or prognosis, or when we seek to identify novel biomarkers, we ultimately care less about hypothesis testing but more about prediction. We demand that a model trained on a set of training data also performs well if we apply it to new observations not contained in our previous training set, i.e., helps with the right diagnosis or prognosis, or with determining the best form of therapy. In other words, the goal is to select the model which will minimize the error when predicting outcomes for unseen individuals based on the learned relationship in the training data. As with the criteria used for model training, the PE may be based on different types of loss functions, for instance the MSE loss or a likelihood-based criterion. There are at least three different types of prediction we need to distinguish (see Fig. 3): The in-sample PE refers to the situation where we keep one part of the data fixed, e.g. the predictors, and aim to determine the expected deviation between a new set of true and predicted outcomes for this given set of predictor values (Fig. 3c). A more interesting quantity is the out-of-sample PE, where we train the model on some data and then draw a new sample to evaluate the PE (Fig. 3c; [28, 54]). In this process, we often assume that the new sample has the same statistical properties as the training sample, i.e., was drawn from the same probability distribution. This may not be the case, however, with important implications for clinical practice. Here we call this the ‘out-of-domain’ PE (Fig. 3d), which can only be determined realistically if we have data from different domains, or if we have a good mechanistic model of the processes underlying our sample.

When we ask ‘how big is big enough?’, we are really asking how large a sample should be, and which properties it should have, in order to be able to infer a model with acceptably low PE. Except for the simplest types of statistical models, like linear Gaussian models (GLM), we cannot simply compute the sample size required to achieve a given PE, since the probability distributions and expectation values involved in this computation are analytically intractable. This is because in ML we are usually dealing with (highly) nonlinear models and consequently more complex probability distributions. Alternatively, one may think of numerical sampling (Monte-Carlo) techniques to evaluate the required expectations, but even these are often out of the question since in machine and specifically DL we are commonly dealing with such high-dimensional variable and parameter spaces that sampling is not computationally feasible [55]. So explicit determination of required sample sizes is not possible for most problems of practical relevance, but what we can do is try to obtain a PE estimate for a given model.

### Bias-variance trade-off and model complexity

**Fig. 4:**
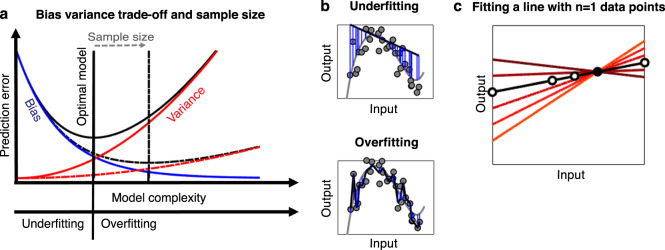
Model complexity and the bias-variance trade-off. **a** As model complexity is increased (*x*-axis), variance rises and bias declines, that is, lower bias is traded for higher variance. We want to select the (optimal) model which balances these two quantities, achieving minimum prediction error (*y*-axis, minimum of bias plus variance, black curve). Increasing sample size effectively shifts this minimum to the right (dotted lines), enabling models of higher complexity. **b** Illustration of underfitting (top) and overfitting (bottom). Both panels depict the same samples (gray dots) drawn with noise from the true function (gray). The low and high complexity linear regression models with polynomial basis expansion of order 1 (top) and 20 (bottom) were fit (thick black lines). Panels depict the model deviation to the true function (blue lines) illustrating model bias. **c** Overfitting in detail: Here we assume that the true relation between inputs and outputs is perfectly linear as depicted by the black line (with 5 data points on that line illustrated). Assuming we have only observed one data point (black solid circle), we can however fit infinitely many lines (some of them illustrated in color) equally well. In this simple example, increasing the sample size just by one data point (and assuming there is no noise in the data) will allow us to pick out the correct model.

Why is the training error, which we can compute directly from the sample at hand, not a good measure for the quality of our model? For a given model with a given number of parameters it indeed is, and so it is completely justifiable to determine model parameters such that the training loss (e.g., the negative likelihood) is minimized. However, it is not a good estimate of the loss we could expect when applying our model to a new sample, and hence is not suitable for selecting among different models with different numbers of parameters. Reasonably complex models such as polynomial basis expansions or multi-layer NNs can fit (i.e., approximate) any function, and hence any given set of training data, to an arbitrary degree, making the training error in fact zero for a sufficient number of parameters (see Fig. 4b, c). This is even true for simple linear models if the number of predictor variables and parameters are as large as or larger than the number of observed outputs (simple example: if you have observed just one predictor/ output pair $\{ x,y\}$ and consider the linear model $y$ = *β*_0_ + *β*_1_*x*, then you can find infinitely many solutions for parameters $\{ \beta _0,\beta _1\}$ that result in an exact fit with zero error; however, only one of these will be the one that describes the true relation between predictors and outputs in the population, as illustrated in Fig. 4c). A regression model with the same number of outcomes as predictors, or with a sufficiently large number of parameters in a reasonably powerful model, can produce a curve that goes through every single data point. At some point such a model will capture the entire variability in the data including noise, implying that it will ‘interpret’ pure noise as systematic and meaningful fluctuation. This phenomenon is also known as overfitting. Overfitting implies large variance in the predictions as each time we draw a new sample, we will obtain a new model, as illustrated in Fig. 4c.

In classical hypothesis testing, we assume that we have a reasonably accurate model of the data to begin with, and perform all probability calculations under this assumption (Fig. 1). In many empirical situations, and particularly in psychiatric research, this approach has not brought on the desired progress [27, 56]. Recently the focus has therefore shifted toward adopting ML approaches to infer more complex models directly from data (see e.g., [9, 27, 31, 56–58] for reviews). A complex model is capable of learning a broader range of functional relationships between features and outcomes (Fig. 2b), and is therefore more likely to fit the training data well (see Fig. 4b). In statistical terms, the model will exhibit low bias, by which we mean the systematic deviation between the true data-generating function and the best possible model estimate of the function, i.e., $( {f( x ) - {\mathrm{E}}[ {\hat f_\theta ( x )} ]} )^2$. For squared error loss and identically and independently distributed (i.i.d.) data, the expected test error can be precisely decomposed into this bias, the above mentioned variance, and an irreducible noise term [54]. One can roughly think of model complexity as a measure of how versatile and flexible a model may align to the data (sometimes referred to its capacity [36]; see Fig. 2b and Fig. 4b). Ideally, we want to select a model as powerful and flexible as possible, yet balancing bias and variance in an optimal manner. This is where sample size considerations and ‘big data’ come in. Larger data sets enable learning ever more intricate relationships from the data, as they allow for more complex models with lower bias while keeping the variance down (Fig. 4a). Sample size effectively shifts the trade-off between bias and variance such that more complex models could be inferred without compromising the PE (see Fig. 4a).

While the bias-variance trade-off is a core concept in traditional statistical learning theory and determines model selection, recent empirical observations with DL models surprisingly suggest that once models are strongly over-fit, beyond the point where a perfect match to the training data is obtained, they may actually lead to even better generalization [59–62]. That is, quite counterintuitively, after the test error first reaches a maximum within the overfitting regime (Fig. 4a), it tends to decline again as model complexity is further increased, leading to a ‘double descent’ curve [63]. In this regime all models almost perfectly fit, or interpolate, the training data. However, importantly, this only occurs if a so-called regularization term is included in the optimization function, which leads to ‘smooth’ function fits by implicitly biasing the training process toward simpler models which exhibit a smaller norm [63]. However, the precise mathematical mechanism underlying this phenomenon is still not fully understood (e.g. [61, 64, 65]).

Model complexity moreover, is not that straightforward a concept, and different definitions exist [54]. It is not merely related to the nonlinearity of a model, or the number of its parameters (except if we stay within a certain model class like GLMs). One can easily give examples of two models with equal number of parameters but with different expressiveness in the sense that one can approximate a larger set of functions than the other. Often statisticians talk about the effective number of parameters or degrees of freedom to express the idea that the true degrees of freedom may depend on the functional relationships and constraints involved ([54], although this is in itself a tricky topic [66]). One quite intuitive concept of model complexity proposed in statistical learning theory, is the Vapnik–Chervonenkis dimension, which quantifies how many data points a model function can neatly separate (or ‘shatter’; [67]).

### Model selection

To select a specific model, or its number of parameters, among a larger class of models, we need an estimate of the out-of-sample PE. Attempts have been made to derive analytical formulae to obtain such an estimate [68, 69], but mostly numerical methods based directly on the data need to be used to produce a reliable estimate. Here we will not review this topic in its entirety, but rather focus on a few commonly used methods that illustrate how sample size and model complexity come into play.

Analytical formulae for model selection usually estimate the PE based on the training error adjusted or penalized by a term that expresses the (average) optimism of the training error. The idea is that the training error is an overly optimistic estimate of the expected test error (as noted above), and so by approximating this optimism and adding it to the training error we should obtain a better PE estimate. In fact, the methods around usually only provide an estimate of the in-sample PE (see above), i.e., the error evaluated across new outcomes sampled at the same data points used for training [54].

Popular analytical formulae are the Akaike information criterion (AIC; [68]) and the Bayesian information criterion (BIC). Denoting by *L*_MLE_ the value of the data log-likelihood evaluated at the maximum likelihood estimator $\hat \theta _{{\mathrm{MLE}}}$, the AIC is given by AIC = −2*L*_MLE_ + 2*k*, from which we immediately see how the number of parameters *k* penalizes model complexity. The sample size *N* affects the AIC indirectly through the log-likelihood: For a constant number of parameters *k*, the likelihood term will gain importance as *N* increases (its numerical size will increase) such that the second, penalizing term becomes less relevant. This example illustrates quite directly how data fit (training log-likelihood) and model complexity (number of parameters) are traded off against each other. Unfortunately, these methods often provide only relatively crude PE approximations (e.g., [70, 71]), and AIC and BIC have been observed to overfit and underfit, respectively [72, 73].

Probably the most popular numerical method for estimating the out-of-sample PE, and somewhat of the current ‘gold standard’, is cross validation (CV). In CV one trains a model on a larger fraction of the available data, say 90%, and then tests model performance on the 10% left-out data that have not been used for model training, thus obtaining an out-of-sample PE. In K-fold CV, this process is repeated for each of the *K* = 10 × 10% data fragments in turn [74], i.e., each 10% section is put aside once for testing while training the model on the remaining 90%, in this way making full use of all the data available for both training and testing across ten iterations. The final PE estimate is then the average across all ten runs. Other ratios of training to test set size are of course possible. The extreme case where only a single data point is left out for testing is called ‘leave-one-out’ CV. It turns out that CV is itself subject to the bias-variance-tradeoff with the proportion of left-out test data as a free parameter [28, 54, 75].

CV could be used to either select a model or assess the out-of-sample PE [54, 76], but cannot be used for both simultaneously. When we use *K*-fold CV to determine the model with lowest CV error which we select for further use among, say, M tested models, we need to be aware that its associated CV error will likely be an overly optimistic PE estimate: Using the CV error both for model selection and model assessment represents a kind of ‘double-dipping’ which will lead to an estimated PE lower than the actual one just by chance, as we had M different attempts to compute a CV error [28, 54, 77–79]. To compute a true out-of-sample PE estimate in this case, we should really split the data into three segments, one for training (training set), one for model selection (validation set), and put one aside purely for PE assessment (test set). Alternatively, when data are scarce a nested CV scheme may be applied in which model assessment and selection are carefully separated by an outer (assessment) loop which separates the data into training, validation, and test set, and an inner (selection) loop, which shuffles only the training and validation set [79]. Nonetheless, leaking data used for model selection into the model assessment step is perhaps the most common mistake made in the literature (see also [77]).

If we have too few data, we may not be able to afford a separate test set or just a small test set. The larger the test set, of course, the lower will be the uncertainty about the mean PE estimate, i.e., its variance or standard error (see also [80]). In fact, too small test (and training) samples may be one explanation why we observe a counterintuitive negative correlation between model classification accuracy and sample size across psychiatric studies [81–83]. Large uncertainty in the PE estimate in combination with publication bias may have resulted in the predominant reporting of high accuracy estimates for small samples (apart from reasons related to larger sample homogeneity within small sample size studies).

To summarize, as our sample increases in size, the variance (standard error) of estimated model parameters will decrease. As a consequence, we can afford more complex models which come with lower bias. Where exactly this tradeoff is optimized, needs to be determined for the specific data and class of models at hand by formal procedures like CV. Other properties of the data, like the amount of irreducible noise or the type of distribution the data were drawn from, will also affect the required sample sizes. If the distribution is very broad, multi-modal or with long heavy tails, we may need larger samples.

### Cross-site and out-of-domain prediction

When we obtain an out-of-sample PE estimate, we assume that any new data we would like to use our model on comes with the same statistical/ distributional properties. Essentially this means that all sources of variability in the data need to be the same across samples, that is, we need truly random samples from the whole population to which we would like to generalize. Variability in a feature (e.g., reduction in brain volume) may emerge from multiple sources such as disease heterogeneity (e.g., reduction is not present across all individuals), biological variability (e.g., brain volume is itself quite variable and may even correlate with other confounds such as age), or from measurement noise (e.g., the assessment of brain volume is noisy). Differences in inclusion criteria may constrain disease related or biological variability, while different measurement devices (for example different MRI machines in multi-site studies) may generate systematically different errors across samples and can thereby result in violations of common distributional assumptions (i.i.d., see also Fig. 3). This in turn may lead to one of the biggest dangers involved in building clinically relevant prediction models, as amply illustrated in the recent literature [81–84]: If, for instance, models are trained and tested on data from the same clinical site or group, or a consortium which employs common procedures among its members, the model may learn predictive but disease irrelevant site-specific characteristics [82, 85]. Of course this reasoning also applies to inferences drawn through hypothesis-driven approaches and could explain heterogeneities across studies by differences in distributional properties of the investigated samples.

### Particular challenges for time series and sequential data

Our goals in time series analysis may be twofold: On the one hand, we may just want to extract temporal features from a time series, like the power in different frequency bands or the functional connectivity, which we would then like to use as predictors in a classification or regression model. In that case, assuming we have time series from *N* independent subjects, we could simply proceed as outlined earlier, since the model ultimately used for prediction is not itself a time series model, but a feed-forward model that simply uses features extracted from *N* independent time series as inputs. Often, however, our goal is to forecast a time series, for instance, we may want to predict stock market shares, or a future patient trajectory from medical records with sequential entries across time [86], or from mobile data like various sensors and ecological momentary assessments [87]. In these cases, we have to consider that time series and sequential data come with their own specific problems since consecutive measurements across time are usually highly dependent, violating the assumption of i.i.d. data that underlies most of statistical testing. Due to these auto-correlations (and potentially non-stationarity) in the data, it is not as straightforward to split the data into *K* folds and perform CV [88]. For instance, we cannot just randomly leave out some fraction of data points, since this would destroy the temporal contingencies on which time series models rest (they are built to detect the temporal structure and use it for forecasting). Even if we leave out temporally contingent time series segments, the question arises how to train the model across the resulting temporal gap. Finally, any left-out segment will be highly correlated with other segments, at least with the directly preceding one, implying that it will not constitute an independent test set as is the basis for determining the PE by CV. If time series from *N* different subjects (or reasonably independent trials) are available, we could instead run the same strategy as above and train models on, say, 90% of the subjects, fix the parameters, and test their prediction performance on the 10% left-out subjects. This comes with additional issues that we will only briefly touch upon here: First, time series data in biology and psychology are generated by some larger underlying dynamical system, which we only partially observe [4, 89, 90]. When we apply our trained time series model to new observations, our estimate of the initial condition (which we need to run the time series model) may therefore be highly ambiguous, often implying a prohibitively large variance in the predictions. Second, especially for time series generated by dynamical systems it is in fact an open question, which metric would be most suitable for assessing prediction performance: In a chaotic dynamical system, for instance, temporal trajectories quickly diverge even when we have captured the true underlying system with our model, rendering conventional MSE or likelihood-based measures directly evaluated on the time series unsuitable [4, 89–91].

### Model training, computational efficiency, and searching complex optimization landscapes

Another point to consider is computational and numerical issues involved in inferring statistical and ML models from data. Unlike simple statistical models for which analytical or straightforward and fast numerical procedures with unique solutions often exist, the optimization landscapes for many ML algorithms, DNNs in particular, may be highly complex, high dimensional, and rugged, such that optimization becomes a serious challenge. Potentially, even if we knew that model A would in principle be the one that optimizes the bias-variance-tradeoff for a given data set (Fig. 4a), the specific point in its multivariate loss function that optimizes this tradeoff may be extremely difficult to find in practice, requiring a lot of computational resources. In general, more complex models take (much) longer training times, and while on the one hand side, big data may be required to sufficiently specify some complex model, on the other hand, they come with a particular computational burden especially for complex models. Hence, one needs to be aware that the applicability of more complicated models like DNNs is not merely limited by the sample sizes required to meet the bias-variance-challenge, but also by the additional issues involved in finding a near-optimal solution (which is usually not that unique in DNNs, see [92]), and the computational costs that come with it.

In sum, regression and classification problems in psychiatry will likely require learning complex mappings between features and outcomes, to integrate across data from multiple domains, and to combine both temporal and spatial information. However, sample size may curtail inferring models of the required complexity by virtue of the bias-variance tradeoff and computational issues involved in both finding minima of loss functions for complex models and in computing hardware and temporal resources.

## DL from big and small data in psychiatry

While DNNs are capable of revealing complex but highly predictive feature combinations, they commonly have a large number of parameters, somewhere between hundreds into the many millions. From the discussion in the previous section one may deduce that for such models a huge amount of data is needed to battle the bias-variance trade-off. For instance, in image processing tasks where DNNs with eight hidden layers and over 60 million parameters (and more units) are commonly employed, over 15 million labeled images are used for training [14]. These are sample sizes which are just not available in psychiatry, in particular when expensive and laborious techniques, such as neuroimaging during cognitive tasks, are involved. However, this does not mean that we cannot employ DNN-based methods in psychiatry. A DNN framework for data analysis consists of (1) the model architecture, (2) a loss function, (3) a training algorithm, and (4) the data itself on which the DNN is to be trained. We can in fact tune all four of these components to make DNN approaches applicable to small or medium sized data sets, as will be briefly reviewed next.

### Network architecture

The specific network architecture determines which class of functions can be computationally efficiently approximated [36]. Choosing a suitable model architecture, by easing the training process, may therefore help to reduce the demands on sample size. In a sense, we are making use of prior domain knowledge to offset potential limitations of the data, a similar strategy as in Bayesian approaches to model inference.

CNNs are an example of networks deliberately designed for processing image information in computer vision [36]. Inspired by the primate visual system [12], CNNs are set up to exploit spatial invariances in an image for extracting feature maps, using units with spatial ‘receptive fields’ (i.e., localized spatial filters). Each feature map is learnt by combining information across multiple receptive fields, using the same set of shared connection ‘weights’. This weight-sharing principle, exploiting the insight that images could be decomposed into features reoccurring at multiple spatial positions, substantially reduces the number of to-be-trained parameters. CNNs are probably the most popular class of DNN models in medicine when developing classifiers on the basis of imaging data (see [9, 10] for reviews). In psychiatry and neurology they have been used, for instance, to classify disorders based on anatomical brain images obtained through MRI (e.g., [93]), functional brain images or measures derived from it like functional connectivity (e.g., [94–96]), or for combining structural and functional neuroimaging data (e.g., [97–99]).

Similar to what CNNs are for the visual domain, in the temporal domain LSTMs are specifically ‘engineered’ systems that enable to extract long-term dependencies in the time series through special ‘memory cells’ and multiplicative gates, which control the information flow into and out of these memory cells [15]. In psychiatry, LSTMs have for instance been used to forecast depressive and manic states in bipolar patients based on mobile data [26], to detect mental disorders from speech [101–103], to discriminate between psychiatric patients and healthy controls [104, 105] (see [106] for GRU based approach), or to process text passages from social media platforms to identify subjects at high risk for drinking alcohol [22].

Another central property of the NN design is the specific form of the units’ activation function (Fig. 2c top). For instance, for many if not most problems rectified-linear unit (ReLU) activation functions represent a particularly efficient choice because they facilitate the training process for specific mathematical reasons (see below, sect. on training algorithms). Luckily, the UAT holds for ReLU functions as well [107]. In fact, the choice of activation function, ReLUs in particular, may be more important than any other network design feature [36, 108].

### Choice of loss function and regularization techniques

The choice of loss function is primarily determined by the scale level of the data (e.g., continuous, ordinal, or categorical data), and by whether we are working within a statistical framework or in a more deterministic ML framework. In ‘conventional’ deterministic ML we often simply go with the MSE criterion, which may be interpreted as a Gaussian log-likelihood under the assumption of a constant identity covariance matrix (thus yielding no insight into true uncertainty). In a statistical framework, we usually desire to model distributions across data which comes with measures of uncertainty, and hence use likelihood-based criteria or Bayesian approaches for model training. Bayesian criteria come with special benefits that affect required sample sizes, but they also make model training more tedious such that most NN optimization is based on likelihood-based approaches (in ML often phrased in terms of the negative log-likelihood, also referred to as cross-entropy for categorical data). However, a statistical approach often entails that we have to treat the network’s hidden activation states as random variables as well, so-called latent variables [109], which implies that we often can only use approximations to the log-likelihood such as the so-called evidence lower bound [55, 110, 111]. While statistical approaches and criteria often take (much) longer for model training, they provide full probability distributions across the data and, in fact, may capture important relations within the data much better [36].

Besides the general consideration of whether we would like to work in a statistical or a deterministic ML framework, the loss function may be modified in particular ways to encourage the training algorithm to find solutions, which reduce the effective number of parameters or model complexity in a specific way. This is called regularization, and more generally has been defined as ‘any modification we make to a learning algorithm that is intended to reduce its generalization error but not its training error’ [36]. The most popular techniques are L1 and L2 regularization, the latter also known as ‘weight decay’, ridge or Tikhonov regularization. L1 regularization adds the sum of absolute parameter values, $\lambda {\sum }_{j = 1}^P \left| {\theta _j} \right|$, to the loss function, while L2 regularization adds the squared parameter values, $\lambda {\sum }_{j = 1}^P \theta _j^2$, where the weight *λ* > 0 controls the relative importance of the regularization (or penalty) term in the loss function, e.g. ${\rm{Loss}} = - \log p\left( {{\mathbf{X}}|{\mathbf{\theta }}} \right) + \lambda {\sum }_{j = 1}^P \left| {\theta _j} \right|$ for an L1 penalty added to the negative log-likelihood. L1 and L2 regularizations are also often used even for simple linear regression models, where the former is known as the ‘least absolute shrinkage and selection operator’ [112], and the combination of L1 and L2 regularizatiosn has been termed ‘elastic net’ [113]. For high values of λ, model parameters will be forced toward 0, where the L1 penalty will make some of them exactly 0 eventually (hence they drop out from the model), while L2 regularization on the other hand tends to shrink parameters associated with features which show low covariation with the outcomes (see e.g., [33, 114] for examples in psychiatry).

L1 and L2 regularizations are common in all types of statistical and ML models, including FNNs and RNN. For DNNs, however, also more specific techniques have been developed to prevent overfitting and encourage sparser and less complex solutions. One particularly effective method is parameter dropout. Here, a fraction of units is temporarily removed at random from the network (e.g., by multiplying their output with 0 and effectively dropping them from the loss function) such that only a ‘thinned’ network is trained at any one time [115]. For testing, all units are then reinstated with their outputs weighted by the probability with which these units were present during training. Dropout seems to drive units to learn more robust representations [115], is computationally inexpensive, and works for both FNNs and RNN [36]. Additional more purpose tailored regularization solutions have been proposed to solve the long-term dependency problem in ‘vanilla’ RNN, e.g., by directly modifying the loss function (e.g., [89]) or indirectly via specific parameterizations of the weight matrices (e.g., [117–118]). Importantly, many of these approaches achieve performance comparable or superior to that of LSTMs with often far less parameters, and are on top more easily interpretable, for instance in terms of the underlying dynamical system and its properties. An intuitive understanding of how networks represent information, e.g., how RNN store memory and implement dynamical systems, can sometimes guide such task-tailored regularization schemes. For instance, by regularizing only a fraction of RNN parameters toward line attractor configurations, we can force a network to store both short and long-range dependencies [89]. Recovering features encoded at both fast and slow temporal frequencies is imperative to identify signatures of aberrant brain function, as different frequencies carry different information (e.g., α or γ-waves in EEG [119]). Likewise, mental health related features inferable from mobile devices and sensors can be found at both fast and slow frequencies (e.g., typing dynamics [24] vs. sleep-wake cycles [120]). Regularization approaches gain importance as sample size decreases and can remarkably improve generalization, as seen for instance in terms of improved dynamical system reconstruction [89].

### Training algorithm

**Fig. 5:**
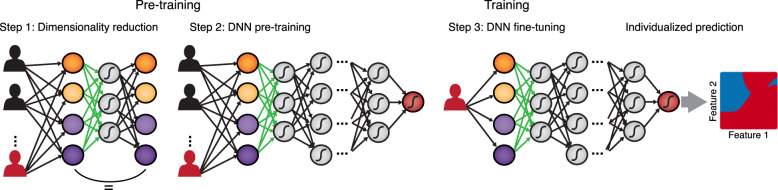
DNNs for individualized (treatment) predictions. In order to employ more complex FNN or RNN models for person-tailored predictions, we can pre-train a NN on multiple individuals. We first reduce the input dimensions, e.g., with an autoencoder (step 1), and then pre-train a DNN on the reduced inputs for a large sample (step 2). The pre-trained network may then be fine-tuned on the specific individual in a third step (right panel). Future data points could then be used to forecast symptom onset, treatment response, or other mental health-related variables.

We have already identified the training (optimization or inference) algorithm as another potential bottleneck that could also affect the required sample size. There are several steps that we can take to improve finding an acceptable local minimum or solution. First, any training algorithm begins with an initial draw of parameter estimates, also referred to as initialization. Naively, one may think of randomly sampling initial parameter estimates which representatively cover the entire (high dimensional) parameter space, but in most cases this is computationally highly demanding and infeasible. A great deal of research has therefore focused entirely on developing efficient initialization procedures in DNNs [36]. Hinton et al. [121] introduced a clever training technique in which layers are pre-trained one after another such that network parameters are already sensibly initialized before the full training of the whole network (see Fig. 5). While this sounds like a rather minor modification, this insight contributed strongly to the groundbreaking success of DNN algorithms. Another larger body of research focuses on so-called annealing approaches [122, 123]. Here, the loss function is gradually modified throughout training such that the training algorithm is first guided into regions of parameter space where generally higher likelihood (lower loss) solutions are to be found, which are then iteratively refined. For example, in so-called Boltzmann machines, a specific type of generative NN model, the ‘energy landscape’ is made initially very flat to encourage the system to escape from local minima, and then gradually steepened, called simulated annealing [123].

As another example, for fully probabilistic models, meaning models which treat both the observations and latent (hidden) variables as random variables, the variational annealing approach proposes to gradually increase the ratio between observation and latent variable noise in the loss function, that is, to decrease the relative noise in the hidden variables across training iterations [124, 125]. The idea is that initiating the latent variable mappings with very high noise (i.e., low precision), essentially makes the optimization criterion (in the limit) a quadratic and convex function of the observations, and thus easy to solve. As the ratio is slowly increased, putting a stronger emphasis on the latent variable model fit, more and more hidden configurations inconsistent with the data slowly ‘freeze’ out. Rather than steepening the overall ‘energy landscape’ as in simulated annealing (i.e. cooling the overall temperature, or variance), this approach gradually decreases the relative temperature of the hidden variable loss.

In addition, the specific procedure by which parameters are updated can have an important impact (cf. section 'Model complexity, sample size, and generalization'). Perhaps the most defining aspects of a training procedure are (1) how it scales with data size and parameters, (2) which information in the data it exploits, (3) which steps it takes to escape local minima, and (4) how it deals with regions of differing slope in the loss function. Perhaps the most popular training scheme for DNNs is stochastic gradient descent (SGD) [126]. The idea behind gradient descent in general is that in order to move toward a local minimum of a function, we simply need to follow steps proportional to the negative gradient of that function. SGD makes use of this principle, but rather than computing the gradient across the entire data set, it computes the gradient from a small subset of (randomly drawn) samples, or mini-batches, thus injecting some noise into the training process that may help to avoid local minima. Particularly for large amounts of data, SGD is computationally efficient and comparatively fast. As noted above, SGD training further profits from certain choices of a neuron’s activation function, like the ReLU: ReLUs have a (piecewise) constant gradient everywhere which eases issues with widely differing slopes in a model’s loss function during gradient-descent training, while sigmoidal activation functions do not only have gradients varying quite strongly across their range of inputs (Fig. 2c bottom), but in particular tend to saturate for very small or very large inputs, making gradient-based training more difficult [36].

Widely varying gradients may, however, also be compensated by taking higher-order information (that is, e.g., 2nd-order derivatives) into account: Through this, approaches such as expectation–maximization or Gauss–Newton methods [28, 109], although computationally (much) more challenging, may be more efficient in finding minima and may thus benefit function approximation based on smaller samples. Another strategy to deal with varying slopes is to adapt the learning rate, a factor with which the gradient is scaled during each step of SGD, locally or across training iterations. Various such algorithms with step-size regulation have been proposed (e.g., Adam or AdaGrad, see also [127]).

Lastly, we point out that modern DNN research has come up with some general procedures to promote model generalizability independent of inference frameworks, which are now part of many standard protocols. These approaches include early stopping or adversarial training procedures (see [36] for more examples). In early stopping, training is stopped when the validation (rather than the training) set error ceases to decrease for some time. The validation error is evaluated every couple of steps and a copy of the associated latest parameter settings are stored such that it can be returned to as training continues [36]. Adversarial training, on the other hand, directly attempts to find weak spots in a model by searching for slight input perturbations which will cause large deviations in the output, and thus could ‘fool’ the model [128, 129]. Szegedy et al. [129] show how such slight (undetectable to humans) distortions of, for example, a dog image can make a NN falsely predict an ostrich. Deliberately searching for and training networks against such flaws renders them more robust to small perturbations around the neighborhood of the training data.

There are also various steps we can take with the data itself to ease the burden on the model side and encourage solutions that better generalize to new observations [36]. One idea is to reduce the dimensionality of the data or pre-process it in some way such that fewer model parameters are needed and the burden on the model training framework to discover most useful representations of the data by itself is reduced. Based on our own domain knowledge, we may preselect features, which we deem to be highly informative. For instance, using the average regional gray matter volume from sMRI images as features will spare the NN the work of identifying and representing distinct segregated regions based on individual voxel values. Most psychiatric studies hand-select features beforehand, for instance, by computing functional connectivity values from the BOLD time series, saving the NN the work of learning which temporal representations are relevant [33], or summarizing crucial genetic information in terms of polygenic risk scores and therefore bypassing the need to locate or detect significant genetic variations or polymorphisms based on the entire genome [130]. In fact, any data processing step may be seen as a kind of feature selection, including preprocessing, rescaling, or selection of regions of interest, since each of these steps involves certain decisions about what is important about the data.

However, the downside of such preprocessing and feature selection based on domain knowledge could be that we overlook important and highly predictive aspects of the data, integrate them away or average them out in some way. In some sense this contradicts the spirit of DL which is supposed to find useful data representations and features on its own [12]. It may therefore be more fruitful to provide the entire voxel level data to DNNs (e.g., [114, 131]).

An alternative and perhaps less biased approach to manual feature engineering could be to ‘automatize’ the process. This may include anything from popular linear dimensionality reduction techniques like principal component analysis, metric or nonmetric multidimensional scaling [28, 109], or simple latent variable models like factor analysis, to nonlinear dimensionality reduction techniques like locally linear embedding [132] and Isomap [133], or more recent methods like ‘t-distributed Stochastic Neighbor Embedding’ (t-SNE) [134] or autoencoders [135, 136]. Autoencoders (AE), for instance, are NNs which project higher-dimensional input data to a lower-dimensional latent space (encoder part), where this lower-dimensional data representation is optimized such that the original input is reconstructed from it with least loss at the output layer (decoder part; Fig. 5, left panel). Hence this whole design can be thought of as a highly nonlinear dimensionality reduction technique that aims to produce a latent representation of most informative nonlinear feature combinations [135, 136]. Gupta et al. [137] were among the first to use an AE combined with a CNN for classification of a neurological disorder. The AE effectively extracted low-level image features later successfully used to assess Alzheimer’s Disease. Pinaya et al. [138] moreover trained an AE to extract features from brain volume data on a large sample of healthy individuals (*n* > 1000). Interestingly, this trained (unsupervised) AE could predict brain volume alterations in patients suffering from schizophrenia or autism (*n* < 100) as compared to control, suggesting that the AE did indeed extract mental-health related features. The approach demonstrates a clever way of making use of (relatively) large already available and openly accessible data sets from healthy individuals for disease classification, or potentially even to gain insight into pathological mechanisms in smaller samples.

Rather than reducing the input dimension, we could also artificially increase the sample size and variation within the sample, an approach termed data augmentation. For image data, this includes rotations, translations, rescaling, flipping, shearing, or stretching of the original images, or simply adding noise (see also [140–141] for speech recognition examples). The idea behind these operations is that they will assist the network in learning invariant, more general representations, robust under certain transformations and conditions where data are only partially observable or noisy. For recognizing a smile on a face, for instance, it should not matter whether the image is blurry, or the face is presented upside down (see [143–144] for examples in neuroimaging and psychiatry).

Another data augmentation strategy involves generative models, that is, models which contain probabilistic latent variables and by virtue of that can—if properly trained—generate data with the same distributional properties as the original data. One such framework that recently became popular for this purpose are so-called generative adversarial networks (GANs) [145]. GANs attempt to approximate the true data-generating distribution by training two networks in competition with each other, a generator and a discriminator network. The generator attempts to create data samples as similar as possible to the true data while the discriminator strives to distinguish true from fake (generated) samples. The two networks co-evolve throughout training, and by attempting to fool the discriminator, the generator, if successful, learns to approximate the data-generating distribution from which new (simulated) data samples may be generated and used for training [146]. Similarly, one could use generative models like GANs to fill in missing values in multi-modal data sets, a common problem in psychiatry, rather than discarding an entire multivariate data point (see e.g., [147]). Along another line, Nguyen et al. [148] used GANs to unbias MRI images from different sites by successfully transforming images from one site into those from another. Such approaches could help to make more efficient use of larger cross-site data sets which often suffer from site-specific heterogeneities. In cases in which we have access to a large unlabeled data set, rather than simulating data, we may also choose to augment the data set by semi-supervised learning approaches such as pseudo labeling [149]. Here a network is first trained on labeled data, then unlabeled data is fed through it to obtain predictions (pseudo labels), and finally the network is trained on the entire (augmented) data set.

Transfer learning is another technique for improving the data situation by transferring knowledge gained in one data domain to a current problem setting that we expect to share some statistical characteristics with the transfer domain [150]. For instance, rather than training a DNN from scratch on an object recognition task each time one faces a new problem setting, machine learners frequently make use of already publicly available trained DNN models like AlexNet [14] or VGGNet [151], and simply fine-tune parameters on their current task. Lu et al. [152] have extended this approach to structural brain recordings and apply AlexNet to identify pathological images. In another example, Thomas et al. [153] train a DNN to decode cognitive states of participants during a working memory task. They demonstrate how pre-training their network on six other unrelated cognitive tasks considerably improves network performance when compared to random initialization. This sort of pre-training or transfer learning saves data resources and training time that would otherwise be required to learn common (often low level) features which the network would have needed to extract anyway (like edges and nodes in an image, [154]). It can also be understood as a feature selection step conducted by another model rather than the data analyst, and that is not fixed but will further be adapted to the current setting through training. There are examples where a CNN designed to classify a neurological condition based on sMRI images has been shown to perform even better when pre-trained on natural images rather than on sMRI data itself, perhaps because natural images are in a sense richer in low-level features also needed to classify sMRI images more effectively [137]. In general, pre-training on any data-rich domain that could be expected to share some statistical distributional properties with the target data set may profoundly help in using complex DNN even for smaller samples. Here, open access data may be of huge assistance. Models could be pre-trained on similar, or ideally perhaps even the same disorder as targeted in a current application, and then merely fine-tuned on the target data (see also [93, 155]).

Pre-training could prove particularly useful when individualizing models for single subjects in order to provide patient-centered (treatment) predictions (Fig. 5). Transferring knowledge already gained from other data sets could prove very valuable in building complex and nonetheless robust individualized models. For instance, we could first train models on data sets across individuals and use the inferred parameters as efficient initializations, which would help fine-tune quite complex predictive models at the single subject level (Fig. 5).

Another way to transfer knowledge from other domains or tasks to the current problem setting is meta-learning. While different definitions exist, meta-learning is most commonly understood as a paradigm where the system ‘learns to learn’, that is, which optimizes the learning process of an algorithm itself via multiple learning episodes or tasks (see [156] for a recent thorough review). For instance, Andrychowicz et al. [157] demonstrate how the optimization procedure of a model can itself be optimized via gradient descent and thereby outperforms handcrafted optimization algorithms in many different settings. The optimization process of the meta-learning algorithm can refer to almost any part of the model, including architecture, parameter initialization, and many more, and may be realized through different optimization procedures such as gradient descent, reinforcement learning, or evolutionary algorithms [156]. One particularly interesting aspect is that such algorithms have succeeded in designing classifiers, which are capable of learning in only a few shots, i.e., from few data instances [158]. It is conceivable that along similar lines pooling multiple psychiatric data sets and using meta-learning principles could yield sets of classifiers which learn quickly on new problem settings.

Many of the approaches discussed, like manual feature engineering, transfer learning, or specific network designs tailored to particular tasks, may be seen as different ways of utilizing prior knowledge to facilitate NN training and reduce the sample size requirements. This aligns with the more general idea in Bayesian frameworks for model training, where previous knowledge is incorporated in a statistically principled way through prior distributions on the parameters [28, 159]. While such approaches come with the danger of biasing the resulting model or parameter estimates in the wrong direction, they are on the other hand known to potentially strongly reduce the variance in the resulting parameter estimates and to protect to some degree against overfitting [160]. It turns out, in fact, that some of the common regularization approaches, like ridge regression, can be derived within a Bayesian approach that places certain priors on the parameters [161]. Hence, Bayesian inference strategies can be used to both import prior knowledge from the same or different data domains into the current parameter estimation, and to regularize models.

In sum, the sample size needed to successfully train a DNN will depend on multiple factors such as the type of data, network size and architecture, type of stochasticity in the data, dimensionality of the feature space, regularization schemes, and the actual target function the DNN is supposed to learn, to name but a few. Power calculations are simply not available for highly nonlinear models like DNNs with complicated likelihood functions and probability distributions, and hence any suggestions regarding sample size could only be based on examples in the literature employing very similar architectures. Perhaps the largest bundle of work to date has been performed in the field of classification based on neuroimaging data. In this field, samples of several hundred participants appear to provide a good starting point for successfully training DNNs, with accuracies roughly around 70% for multi-site studies and binary classification problems (see [162] for a recent review). These results raise hope that future DNN applications may prove valuable for sample sizes available in psychiatry. Samples below *N* = 200 produce very heterogeneous performance results, and usually do not contain data from multiple sites, making it difficult to judge models in this range [162]. Recommendations for sample sizes for other applications are more difficult to provide. In general, we strongly recommend that authors employing DL techniques conduct thorough evaluation on prediction errors and their standard error themselves, e.g., by iteratively increasing the test set size (as done e.g., in [80]).

## Future research directions

Psychiatry is in urgent need of approaches that enable tailored precision therapies. For designing efficient treatments, we also require a better understanding of the neurobiological mechanisms underlying pathology at a transdiagnostic level. While more traditional hypothesis-driven statistical approaches to these issues have not brought the necessary breakthroughs, modern ML algorithms like DNNs provide new hope given their outstanding performance in other medical domains. At first sight, the complexity (and thus computational strength) of DNNs comes at a cost—large sample sizes. However, as we tried to discuss here, there are several ways to make DNNs suitable even for much smaller sample sizes. We have discussed various concrete steps to enable the development of efficient schemes using complex models for individualized person-centered predictions (see also [9, 87]). Models first trained on group data may provide one future avenue (Fig. 5), if it can be achieved that these capture sufficient particularities at the (individualized) single-subject level to yield meaningful forecasts, and not just reflect common group characteristics.

A deeper understanding of hidden network representations in DNNs, i.e. ‘opening the black box’, could on the other hand reveal new insights or generate new hypotheses regarding pathological neurobiological mechanisms. Indeed, several studies have already demonstrated that DNN representations may yield interpretable features (e.g., [33, 94, 99, 163]). For instance, by examining the weights of their DNN, Zeng et al. [94] observed that cortical-striatal-cerebellar functional connectivity features were most relevant to the classification of schizophrenia. After training a deep AE on brain volume data from a large set of healthy individuals, Pinaya et al. [138] assessed the region specific reconstruction error made by the network when predicting psychiatric patients to pinpoint the most relevant brain regions involved in separating patients from controls. Li et al. [163] developed a visualization framework to decipher regions of interest important in the detection of individuals with autism spectrum disorder compared to controls based on fMRI recordings. Visualization approaches for assessing DNNs are currently a hot topic in ML, and future developments in this direction may help uncover interpretable multi-modal biomarkers of psychiatric disease. The interplay between the bench and the bedside, pathophysiological understanding and tailored treatment, continues in the age of AI, aided by the new tools discussed in this paper.

## Funding and disclosure

GK received funding from the German Research Foundation (DFG; TRR265: A06 & B08). AML received funding from the German Research Foundation (DFG; ME 1591/4-1; grant 5485966 Collaborative Research Center 636 subproject B7; grant 255156212 Collaborative Research Center 1158 subproject B09; grant GRK 2350/1 Research Training Group 2350 subproject B2; grant TRR 265 Collaborative Research Center subproject S02) and from the German Federal Ministry of Education and Research (BMBF; grant 01EF1803A, and grant 01ZX1904A). DD received funding from the German Research Foundation (DFG; Du 354/8-2; TRR265: A06; Du 354/10-1). AML has received consultant fees from Boehringer Ingelheim, Elsevier, Brainsway, Lundbeck Int. Neuroscience Foundation, Lundbeck A/S, The Wolfson Foundation, Bloomfield Holding Ltd, Shanghai Research Center for Brain Science, Thieme Verlag, Sage Therapeutics, v Behring Röntgen Stiftung, Fondation FondaMental, Janssen-Cilag GmbH, MedinCell, Brain Mind Institute, Agence Nationale de la Recherche, CISSN (Catania Internat. Summer School of Neuroscience), Daimler und Benz Stiftung, American Association for the Advancement of Science. In addition, he has received speaker fees from Italian Society of Biological Psychiatry, Merz-Stiftung, Forum Werkstatt Karlsruhe, Lundbeck SAS France, BAG Psychiatrie Oberbayern, Klinik für Psychiatrie und Psychotherapie Ingolstadt, med Update GmbH, Society of Biological Psychiatry, Siemens Healthineers. Open access funding provided by Projekt DEAL.
